# Direct and remote induced actuation in artificial muscles based on electrospun fiber networks

**DOI:** 10.1038/s41598-022-16872-2

**Published:** 2022-07-29

**Authors:** Mihaela-Cristina Bunea, Mihaela Beregoi, Alexandru Evanghelidis, Andrei Galatanu, Ionut Enculescu

**Affiliations:** Functional Nanostructures Laboratory, National Institute of Material Physics, Atomistilor Str., No. 405A, 077125 Magurele, Ilfov, Romania

**Keywords:** Actuators, Nanoscale materials

## Abstract

The present work reports a new configuration of soft artificial muscle based on a web of metal covered nylon 6/6 micrometric fibers attached to a thin polydimethylsiloxane (PDMS) film. The preparation process is simple and implies the attachment of metalized fiber networks to a PDMS sheet substrate while heating and applying compression. The resulting composite is versatile and can be cut in different shapes as a function of the application sought. When an electric current passes through the metallic web, heat is produced, leading to local dilatation and to subsequent controlled deformation. Because of this, the artificial muscle displays a fast and ample movement (maximum displacement of 0.8 cm) when applying a relatively low voltage (2.2 V), a consequence of the contrast between the thermal expanse coefficients of the PDMS substrate and of the web-like electrode. It was shown that the electrical current producing this effect can originate from both direct electric contacts, and untethered configurations i.e. radio frequency induced. Usually, for thermal activated actuators the heating is produced by using metallic films or conductive carbon-based materials, while here a fast heating/cooling process is obtained by using microfiber-based heaters. This new approach for untethered devices is an interesting path to follow, opening a wide range of applications were autonomous actuation and remote transfer of energy are needed.

## Introduction

The research field dealing with artificial muscles evolved steadily during the last decade. The growing interest is fueled by the development of application areas such as robotics (an intense quest for soft, animal like robots is taking place nowadays) or medical/healthcare devices^1–7^. New and improved types of such components are developed and reported in literature, the actuation processes exploited being based on a wide range of physical and chemical phenomena. Ranging from electrochemically controlled conducting polymer muscles to pneumatic or thermally activated ones it is easy to note a constant improvement in capabilities^8–14^. However, there is still a long way until achieving similar output, in terms of functionality and efficiency, to either biological muscles on one side, or to mechanical, electromechanical, or pneumatic motors on the other side. Improvements in the field are constantly reported in both the mechanisms of actuation and in the types or combinations of materials. Various materials are investigated such as conducting or piezoelectric polymers, elastomers, shape memory alloys, each with its specific actuation mode, its advantages, and disadvantages^15–22^.

Electrochemically induced structural changes, piezoelectricity, thermal or other intrinsic characteristics are exploited in order to achieve actuation. Dielectric elastomers are among the preferences for fabricating soft artificial muscles because of characteristics which include high elasticity, light weight, and a mechanical response to an applied electric field. However, problems arise due to relatively high voltages needed for actuation and the necessity of employing electrodes with a similar flexibility for achieving a high functionality.

Employing composites containing microscopic particles or structures such as carbon nanotubes and graphene became a widely used path to achieve enhanced properties or new functionalities^2,23–25^. Aouraghe *et* al. proposed an electro-thermal actuator based on a PDMS/carbon nanotube composite film which shows an important bending angle (~ 200°) at a temperature of about 350°C obtained when a relatively high voltage is applied^26^. However, the actuator tkes a long time to recover the initial shape (~ 150 s). Similarly, Sun *et* al. reported the fabrication of an electrothermal actuator based on carbon nanotubes and PDMS that can bend ~ 540° for an applied voltage of 12 V^27^. This actuator also takes a long time to reach the steady state bending angle (130 s). Yao et al. developed a bimorph actuator based on PDMS and silver nanowires (AgNws) which bends 30 mm at a low applied voltage of 4.5 V^28^. However, the actuator requires a relatively long time to reach the maximum degree of displacement (~ 40 s) and to recover its initial position (~ 60 s). Hu et al. proposed another bimorph actuator based on a graphene sponge and PDMS which reaches a maximum bending of 12 mm in a relatively long time (60 s) at a relatively low applied voltage of 10 V^29^.

For mechanical actuating devices the geometry and the structure are essential parameters to achieve the desired movement. A wide range of forms and structures are used ranging from coiled polymer fibers responding to changes in temperature to bilayer and trilayer conducting polymer structures responding to changes in potential or elastomeric structures responding to relatively high electric fields^18,30–32^. Each of the structures mentioned are obviously needing energy to perform the actuation. In most of the times the energy is directly transferred using electric leads from a source to the device as is the case of conducting polymer, piezoelectric or elastomeric actuators. When the actuation is related to thermal dilatation processes, in most cases changes in ambient temperature are the source of energy while in the case of humidity responding actuators, obviously, the source of movement is represented by changes in environmental humidity^14,33^. This generates a set of problems since in some cases it limits the possibilities of employing the actuator in a particular, practical device. This is the reason why an intense effort is directed towards autonomous artificial muscles, where the energy is either stored locally or is wirelessly transferred. Different approaches are used including here biomimetic processes where glucose and specific enzymes are employed, or electromagnetic radiation induces movement by local heating^34–37^.

Electrically driven actuators need electrodes in order to transform the external electrical energy into movement. This is the case for all conducting polymer, piezoelectric and elastomer-based actuators^22^. It is not an easy task to provide a material with a set of efficient electrical contacts since mechanical actuation requires flexibility and adhesion to the substrate. It is rather straightforward to fabricate high quality contacts onto polished, rigid substrates such as silicon wafers, but the task becomes more difficult when dealing with flexible and rough substrates such as plastics, paper, or textiles^38^. This becomes even more difficult when constant, long term mechanical motion is added. For such applications high electrical conductivity and flexibility/bendability are both important prerequisites since the device is expected to perform thousands of movements during its lifetime.

This report presents a new architecture of an artificial muscle based on a gold covered nylon 6/6 microfiber web electrode attached to a thin foil of PDMS. The use of metalized electrospun fibers as electrode is useful for manufacturing soft actuators due to specific features including excellent flexibility, high specific area, good mechanical stability, and a straightforward fabrication process. Electrospinning is a versatile method used to produce ultrathin fibers by applying a high-intensity electric field to a polymer solution or melt droplet. The technique can be used for a variety of polymeric materials, uniform nonwoven meshes being obtained from small amounts of precursors while the properties of the fibers can be tailored depending on the targeted application^39,40^. The use of film based classical electrodes in artificial muscle applications leads to some issues which include low flexibility, high specific weight, and limited specific area for energy transfer. In the architecture described in the present report an electrical current passing through a microfiber web electrode generates local heating and subsequent material expansion. Further this leads to a controllable bending of the device with ample and relatively fast movement. By using microfiber webs with high specific surface as heating elements, fast heating/cooling processes are targeted, decreasing the working voltage, while improving the bending properties of the actuator. Electrical currents heating the fiber webs are obtained either by employing a set of direct contacts or are remotely induced by means of radio-frequency electromagnetic radiation. Thus, another important result described in this report is obtaining bending actuation with contactless induced currents which can open new application fields for this type of materials.

## Results

### Artificial muscle actuation properties

The actuation processes in artificial muscle architectures based on PDMS and metalized electrospun fiber networks (labeled as Au/Nylon-PDMS) were investigated by applying different voltages (0.5, 1, 1.5, 2 and 2.2 V) on V-shaped samples with electric contacts on both V segments. The V shape of the samples was chosen in order to ensure a uniform heating when an electric current passes through the conducting component while one extremity can move freely.

The electric power dissipated through the sample as heat due to the Joule effect was estimated as P_w _= E·I, where E is the applied voltage, and I is the recorded current. All the measurements were performed in air, at room temperature.

**Figure 1:**
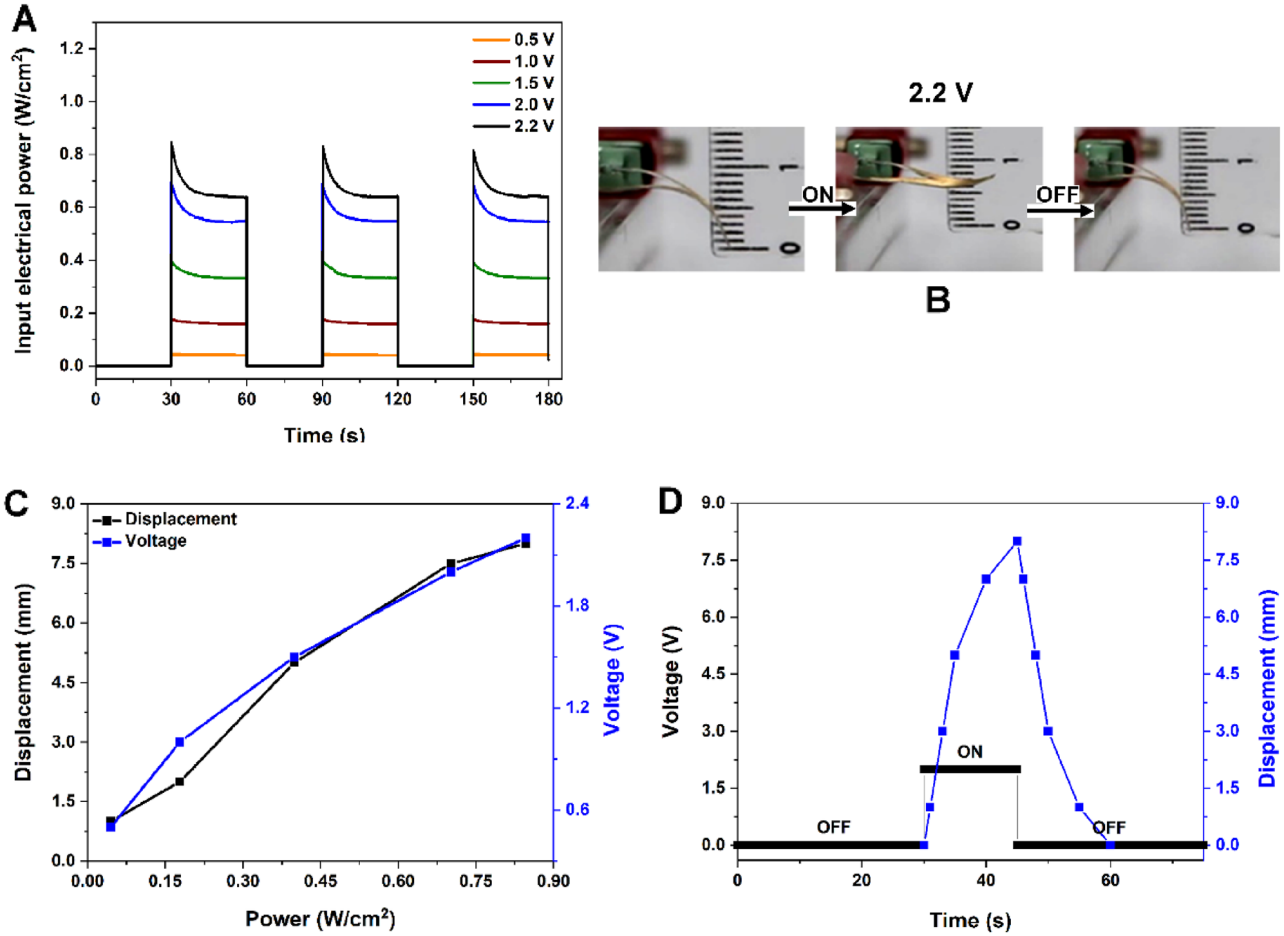
Actuation process: (**A**) the input electrical power versus time and (**B**) snapshots taken during the Au/Nylon-PDMS movement at 2.2 V; (**C**) maximum displacement and voltage versus input electrical power; (**D**) displacement behavior versus one applied ON/OFF voltage pulse.

The measured power for moving the artificial muscle, as a function of time, for each applied voltage, when using *30s on* and *30s off* pulses, is showed in Fig. 1A, while snapshots taken during the actuation process (from actuation Suppl. movie S1) for a complete *on/off* cycle with the maximum applied voltage are displayed in Fig. 1B.

In Fig. 1C a plot of the recorded displacement at the tip of the V and of the applied voltage as a function of the electrical power dissipated through the sample are presented. As it was expected, the amplitude of the movement increases by increasing the applied power (increasing the applied voltage) and therefore increasing the temperature, reaching a displacement of 0.8 cm when applying 2.2 V. When applying a voltage of 2.4 V, the amplitude stops increasing and for higher voltage the fiber electrode becomes unstable and gets damaged.

Data for displacement versus time during a single *on/off* cycle is plotted in Fig. 1D showing that the required time for reaching the maximum displacement is 15 s followed by a period of 30 s for the artificial muscle to return to its initial state and to become able to produce a new movement with the same amplitude.

### Heating processes and thermophysical properties

**Figure 2:**
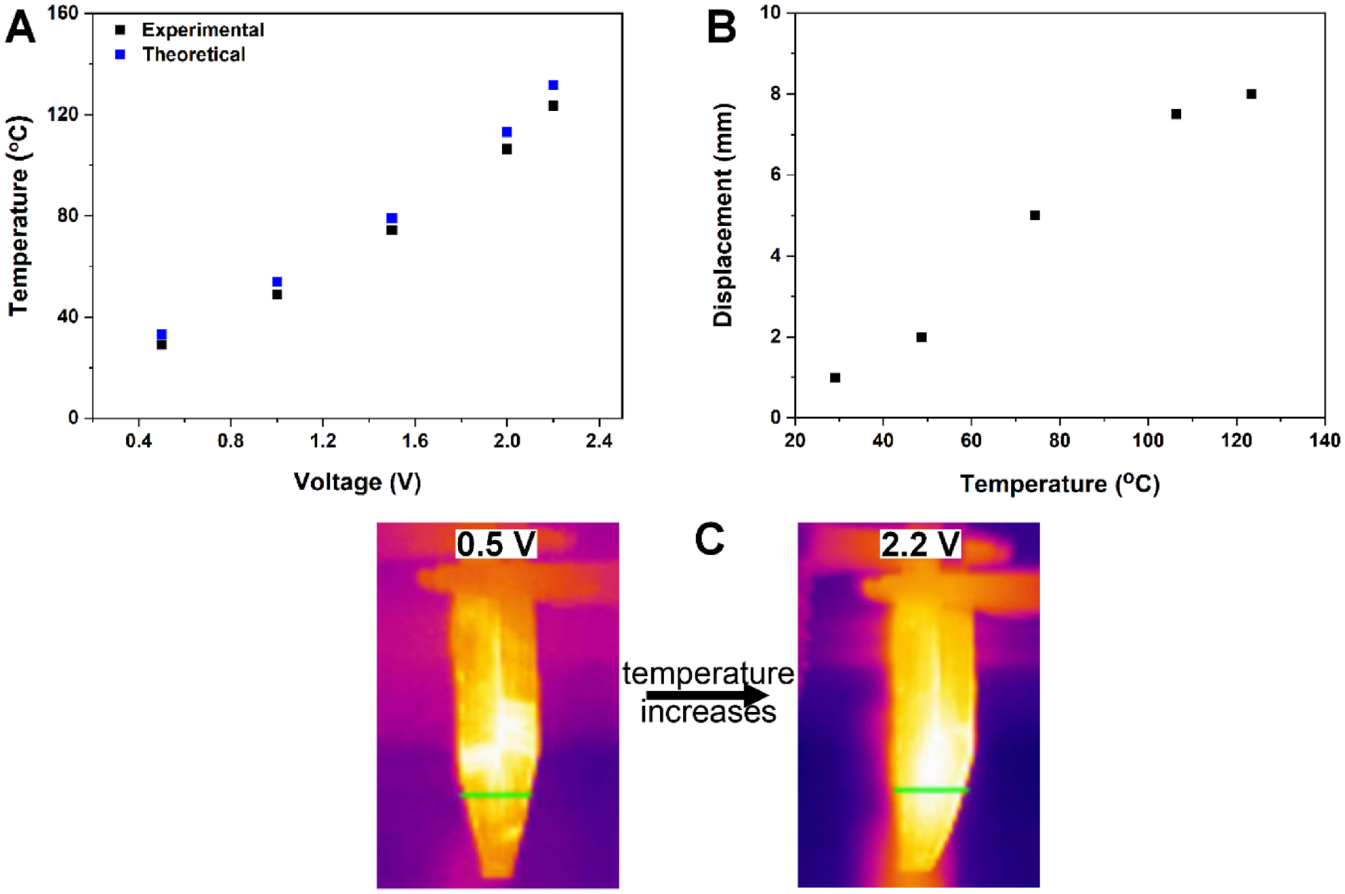
The heating process: (**A**) direct and indirect estimated temperature as a function of the applied voltage and (**B**) tip displacement as a function of local temperature; (**C**) snapshots taken with the IR camera during the artificial muscle heating.

To characterize the heating of the Au/Nylon-PDMS based artificial muscle, a comparison between directly recorded and indirectly estimated sample temperature was performed (Fig. 2A). The indirect temperature estimation was made by using the recorded electrical current, the applied voltage and the following basic equation: R = R_0_[1 + α(T − T_0_)], where R is the resistance at temperature T, R_0_ is the resistance at room temperature (T_0_) and α is the temperature coefficient of resistance for Au (0.0034 °C^−1^)^38^.

The direct measurements on sample temperature were obtained from IR measurements when different voltages were applied on the V shaped artificial muscle. The results show a good agreement between the two methods of estimating the sample temperature. It was found that the temperature of the Au/Nylon-PDMS rises and stabilizes after about 15 s after applying the voltage pulse, the maximal obtained temperature being ~ 125 °C at 2.2 V. This recorded temperature was sufficiently low to prevent the electrical burn of the micrometric fibers. As it was mentioned above, the movement amplitude depends on the temperature until a saturation is reached as can be seen in Fig. 2B. The thermal IR images of the V shaped Au/Nylon-PDMS samples are presented in Fig. 2C and evidence the relative uniformity of the heating process. The fact that a good correlation between the temperature directly measured with an IR camera and the temperature estimated from the resistance behavior exists is important for applications where one can employ only the current for estimating the temperature of the device.

**Figure 3:**
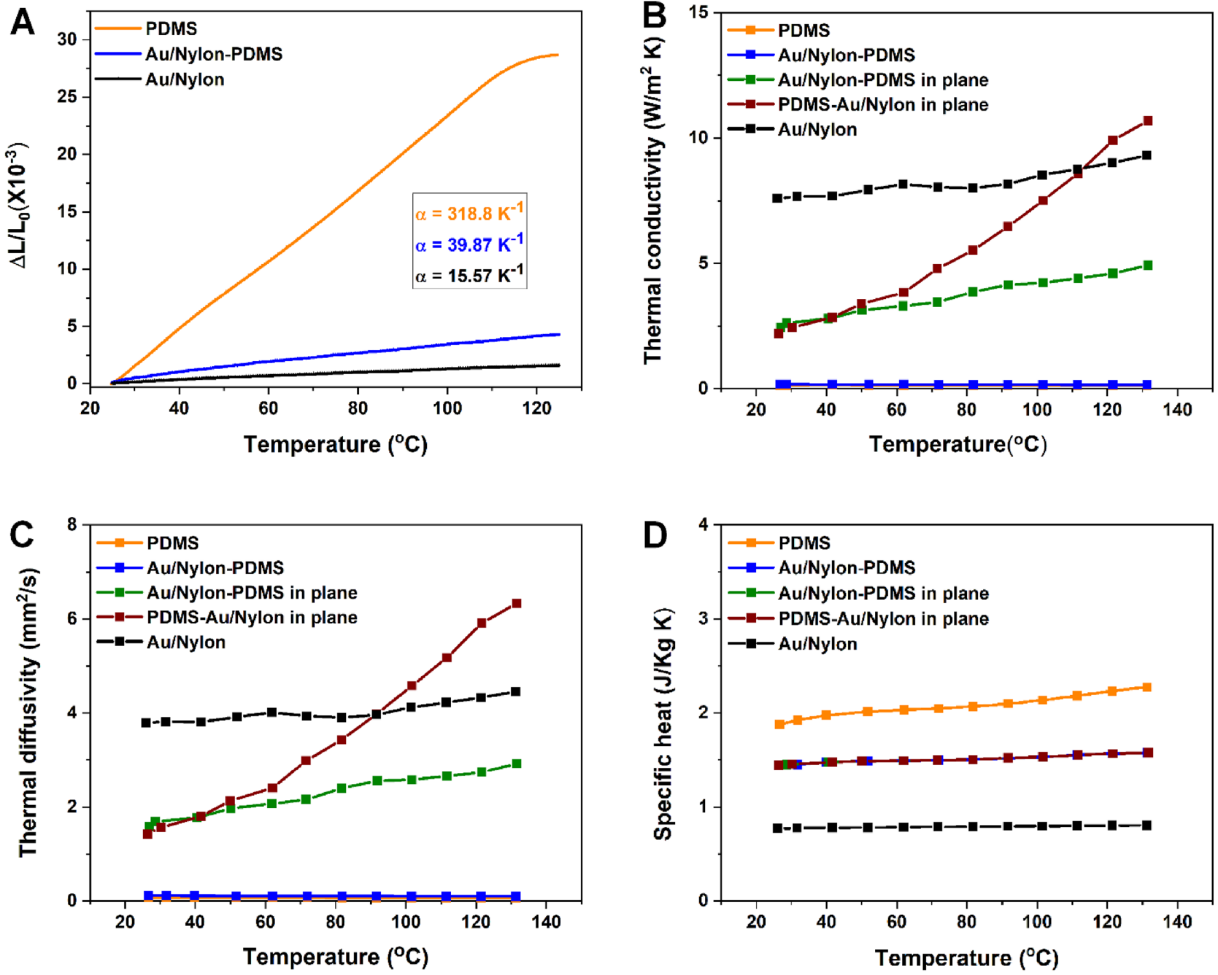
Thermophysical properties of the prepared materials. (**A**) thermal expansion of PDMS, Au/Nylon-PDMS and Au/Nylon measured in plane; (**B**) thermal conductivity versus temperature; (**C**) thermal diffusivity versus temperature; (**D**) specific heat versus temperature.

The thermophysical properties of all the elements part of the architecture i.e. the PDMS layer, the metalized fiber webs (labeled as Au/Nylon) and Au-Nylon/PDMS were measured. The coefficient of thermal expansion, thermal conductivity, thermal diffusivity, and specific heat were measured in the temperature range where the artificial muscle produces a movement, the data being plotted in Fig. 3. The thermal expansion of Au/Nylon-PDMS is presented in Fig. 3A in comparison with the PDMS and Au/Nylon (estimated from the measurements of the first two materials). The thermal expansion of all materials increases by rising the temperature up to ~ 130 °C. As it was expected, the thermal expansion of PDMS is considerable higher when compared to the Au/Nylon system. In the case of the Au-Nylon/PDMS, the thermal expansion shows a slight increase in comparison to Au/Nylon, but the overall behavior is quite similar. The coefficients of thermal expansion of PDMS and Au/Nylon are similar with those reported in literature for bulk PDMS and Au^41,42^.

In Fig. 3B the thermal conductivities of the PDMS sheet, Au/Nylon-PDMS (analyzed out of plane and in plane in two configurations: with either the metallized web or PDMS (labeled as PDMS-Au/Nylon) in front of the heating source) and Au/Nylon (estimated from the first two materials measurements) are plotted. As it was expected, the thermal conductivity of the PDMS sheet does not depend on the annealing temperature, its value being similar to that reported in literature for bulk PDMS^43^.

The out of plane Au/Nylon-PDMS has similar thermoconductivity to the PDMS sheet, while the Au/Nylon system presents the highest thermal conductivity because of the presence of the metallic layer. In the case of Au/Nylon-PDMS, different values of the in plane thermal conductivity are obtained if the metalized web is placed facing or if on the opposite side of the heating source. In both configurations, the thermal conductivity increases by rising the annealing temperature, the PDMS-Au/Nylon one reaching a similar value with that of Au/Nylon.

The thermal diffusivity measurements were used to determine the rate of heat transfer of PDMS, Au/Nylon-PDMS and Au/Nylon (estimated from the first two measurements). The obtained results are presented in Fig. 3C. In all cases, the thermal diffusivity is in direct proportion with the thermal conductivity, exhibiting the same behavior, namely: the PDMS, out of plane Au/Nylon-PDMS and Au/Nylon thermal diffusivities do not depend on the annealing conditions while the position of the heating source and the temperature strongly influence the in plane thermal diffusivity of Au/Nylon-PDMS and PDMS-Au/Nylon.

Another important characteristic of the thermal analysis is the specific heat presented in Fig. 3D for the PDMS sheet, Au/Nylon-PDMS and Au/Nylon (estimated from the first two materials measurements). The specific heat of the prepared materials exhibits a natural trend, thus the PDMS has the highest specific heat, while the Au/Nylon the lowest one. The Au/Nylon-PDMS measurements regarding the specific heat were not dependent on the position used with the metal covered web facing the laser or on the opposite side. In all cases, the specific heat does not depend on the annealing temperature.

### Theoretical modelling

**Figure 4:**
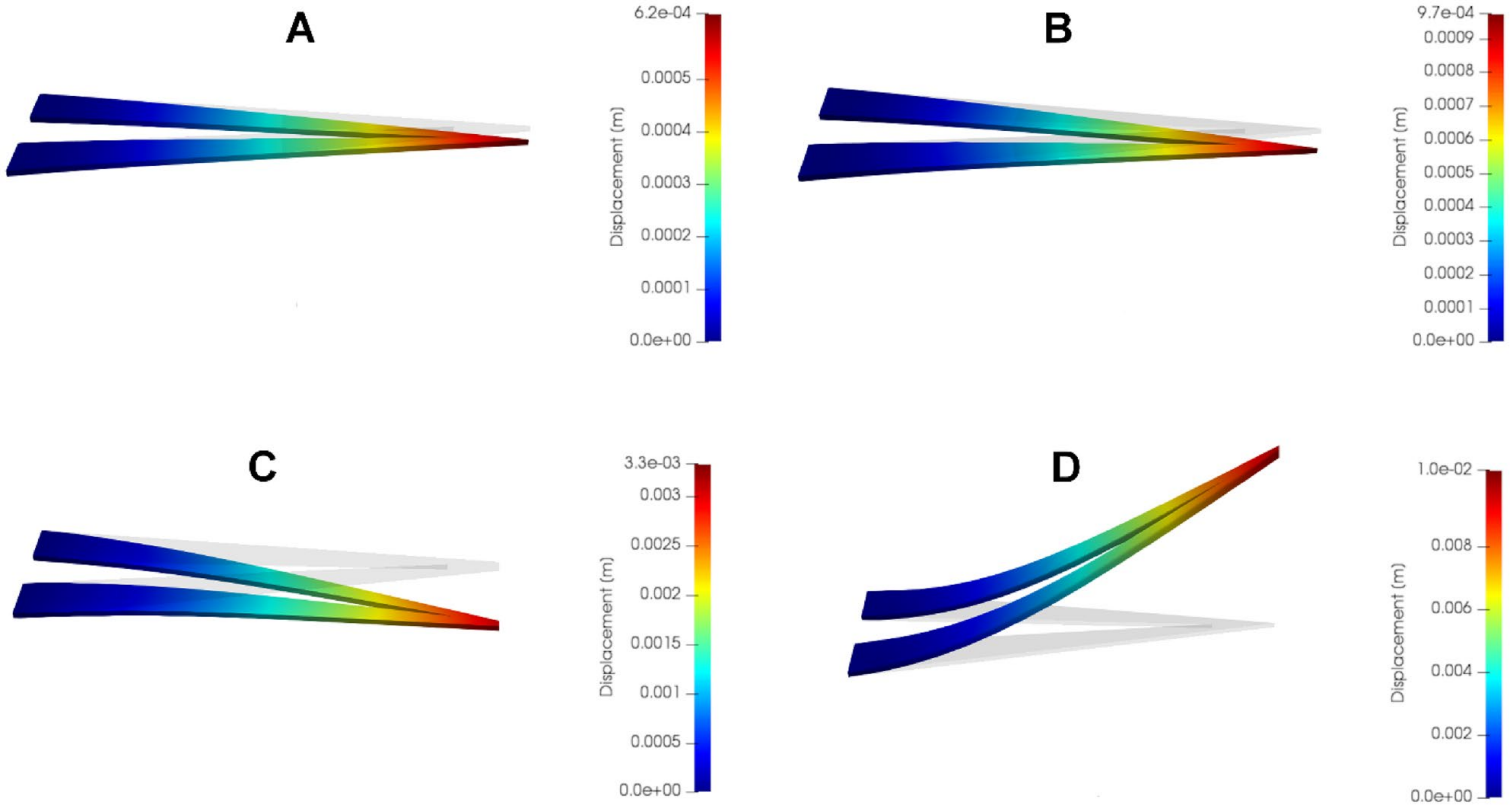
FEM results of the actuating process (**A**, **C**) with and (**B**, **D**) without mechanical involvement from the fibers, in (**A**, **B**) off state and (**C**, **D**) on state.

In order to better understand the mechanisms through which the actuation is generated, a finite element model of the artificial muscle was created, and two hypotheses were tested and compared with the experimental results (Fig. 4). The first hypothesis was that the movement is generated due to a steep temperature gradient being created in the PDMS sheet, which is a good thermal insulator, leading to a differentiated expansion between the fiber covered top layer and the lower layers. Such a variation in expansion would lead to buckling and, therefore, movement. In this hypothesis, the metalized fiber layer was presumed to have no impact on the mechanical properties of the structure. Contrariwise, a second hypothesis was tested, in which the metalized fibers are treated as a separate body with different thermo-mechanical properties, thus creating a bilayer structure. A V-shaped 3D model of approximately the same dimensions as some of the physical artificial muscles (aprox. 250 μm thickness, 2 cm length, 200 nm thickness for the metalized fiber layer if applicable) was created using the Salomé-Meca suite (https://www.salome-platform.org/). Material properties were taken from literature^44^ and steady-state, linear, isotropic conditions were considered for both the thermal and mechanical stages of the simulation in the first, single-layer hypothesis, while for the bilayer hypothesis, only the thermal stage was isotropic.

The metallic electrospun fiber layer was instead defined as an orthotropic material with a much lower elastic modulus along the normal axis (7900 times lower than the in-plane values), to account for the fibers’ excellent bendability. The shear modulus was also set to a negligible value, to account for the discontinuous nature of the layer.

The temperature of the fiber layer (or imposed by it) was set to 443 K (150 °C), the maximum temperature experimentally recorded, and a heat exchange boundary condition was set for all the non-fiber and non-contact surfaces. A relatively high heat transfer coefficient of 1000 W/m^2^ K to approximate the faster cooling due to the micro-structuring of the layer. Gravity was considered as acting upon the body, and simulations without voltage applied were also run.

In the first hypothesis, where the fibers were not mechanically relevant (Fig. 4A), the simulation led to a moderate deflection on the Z-axis, but in the opposite direction compared to the experimental results, i.e. in the same direction as gravity. A considerable elongation along the X-axis is also noticed. The second hypothesis led to much more realistic results, with Z-axis deflections of similar scale to the observations and in the right direction, as well as without any elongation (Fig. 4B). Following these results, a further simple experiment was performed, in which an actuating architecture and a simple piece of PDMS were placed directly on a hotplate, without electrical contact. The actuator’s fiber layer was initially facing away from the hotplate and afterwards it was facing down, towards it. In both cases the actuator begun bending inwards, towards the fiber, while the PDMS layer did not show any bending.

### Artificial muscle lifetime

**Figure 5:**
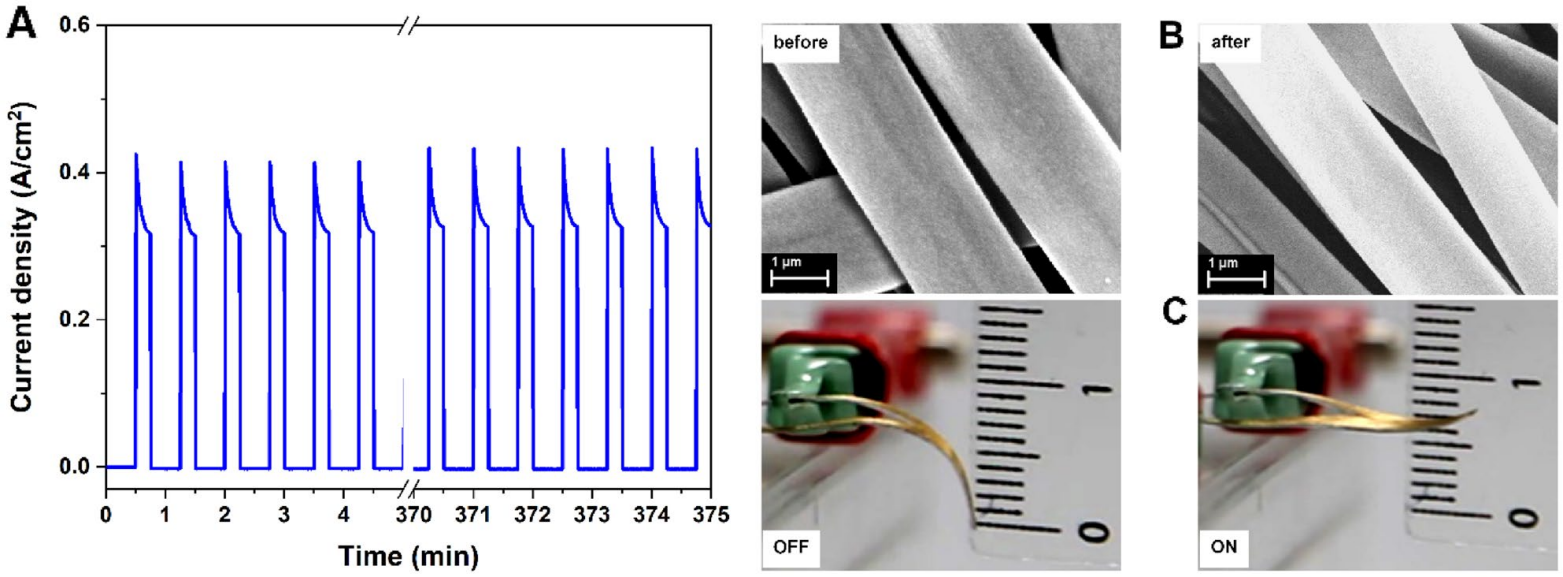
Artificial muscle life time. (**A**) The current density registered for 500 actuation cycles; (**B**) SEM images of the metalized electrospun fiber network attached on PDMS sheet before and after 500 ON/OFF voltage cycles; (**C**) Snapshots taken after 500 actuation cycles of the artificial muscle.

To evaluate the stability of the PDMS based artificial muscle a 500 cycles test performed while applying a voltage of 2.2 V (corresponding to the maximum displacement) was performed (Fig. 5). The tested device kept the same characteristics during the entire duration of measurement showing a good stability over time, as one can observe in value of current plotted in Fig. 5A. The artificial muscle stability is further supported by the SEM images (Fig. 5B) and by the photographs taken after 500 *on/off* cycles displayed in Fig. 5C.

### Contactless actuation of the artificial muscle

**Figure 6:**
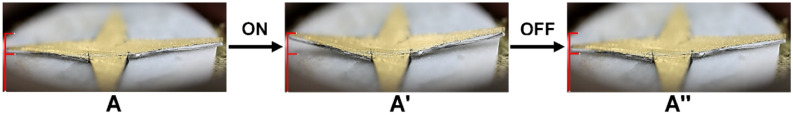
Snapshots of remote actuation by applying a rf field.

The possibility of achieving functional Au/Nylon-PDMS architectures was further investigated by using different shaped objects (Fig. 6). Radio frequency heating tests were performed using a “star” shaped device which was placed in the coil of an induction heater. Its bending movement takes place in a similar manner to direct contact samples, when the coil induction heater is turned *on* (Fig. 6A’) the sample bends upwards, for *off* (Fig. 6(A’’) relaxing to the initial shape, the presented snapshots being taken from the Suppl. movie S2. A displacement of about 1 cm was recorded, the movement being fast, the device returning to its initial position when closing the heating source.

The movement of the artificial muscles described in the present paper is similar to the bending movement of living organisms, noting both their reversibility and their repeatability. The tested devices were able to support numerous actuation cycles without any evident damages. According to our knowledge, actuation using remote energy transport by means of RF electromagnetic field has not been reported so far in the scientific literature and can lead to the development of devices with enhanced actuation properties which do not require any electrical connections.

## Discussion

The present work describes an actuating architecture based on micrometric fiber heaters and a PDMS film. The bending—unbending mechanism is related to the expand/contract cycles which take place during the heating/cooling achieved by applying electrical power either directly or by an RF electromagnetic field. A good control on the movement based on the applied electrical power can be achieved, the displacement depending on the temperature of the microheater system. At a certain applied voltage, the system reaches a maximum bending angle, and a further increase will not generate any further displacement. Moreover, the actuating cycles are relatively fast meaning that the device is heating/cooling rapidly due to the microstructure of the heater (Au/Nylon fiber webs). The notable actuation properties obtained for this Au/Nylon-PDMS architecture are mainly a consequence of the large difference between the coefficient of thermal expansion of the substrate (PDMS sheet) and that of the microheater (Au/Nylon web). When PDMS heating takes place, the movement of the PDMS chains is induced, the molecular chain presenting a dilatation due to the diffusion motion of the chain segment^45^.

It is important to note that the heater’s temperature directly recorded with an IR camera and that determined indirectly by using the electric resistance are in concordance, giving a precise parameter, microheater’s resistance, which can be monitored in order to adjust the desired movement range.

The properties of this architecture are strongly dependent on both the properties of the microheater (electric resistivity, thermal conductivity, dimensions) and on the properties of the PDMS layer, leading to a bending actuation in the microfiber mesh direction.

Connecting the results of the FEM modeling with direct experimental observations, it can be concluded that the mechanism of movement for this actuator is a type of bilayer actuation similar to biomechanical muscle-bone systems, where the PDMS acts as the muscle, expanding and contracting, while the metallic fiber layer acts as a relatively stiff network that guides the expansion/contraction in a way which creates useful movement. This opens the possibility of creating a simpler modeling workflow, like those that are being developed for larger-scale soft robots, allowing complex actuating machines based on this PDMS/metallic fiber material to be tested virtually before physical fabrication.

The 500 actuation cycles test shows an artificial muscle stable over a relatively long period of time, meaning that no damage of fibers occurs during opperation and the process is reversible. This is correlated with the stability of both the components i.e. the PDMS sheet and the microfiber web.

Summarizing, an artificial muscle which produces bending movement with ample displacement and good mechanical stability while applying electrical power through direct contacts or an external RF field was fabricated. The functionality is based on combining the flexibility of the electrospun nylon fiber-based electrodes with the elasticity and deformability of PDMS sheets. It was found that by using metalized meshes as electrodes, the heating/cooling processes are fast and induce the actuation process. The high coefficient of thermal expansion of PDMS in contrast with the lower value of this coefficient for the metalized webs represent the source of the bending movement. All the experimental measurements were in a good agreement with the modeling of how the actuation is produced through the contrast in properties between the two components. In order to remove the necessity for electrical contacts, the fabricated artificial muscle architecture was tested by achieving heating through applying an RF electromagnetic field, demonstrating also for this case a relatively fast and reversible bending movement.

## Materials and methods

Sylgard 184 Silicone Elastomer Kit (Dow) was used for fabricating PDMS sheets, while nylon 6/6 (Aldrich) and formic acid (≥ 88 %, Sigma-Aldrich) were utilized in electrospun fiber meshes fabrication process. A gold target with 99.99% purity from Kurt Lesker was used for metalizing the fiber webs.

**Scheme 1:**
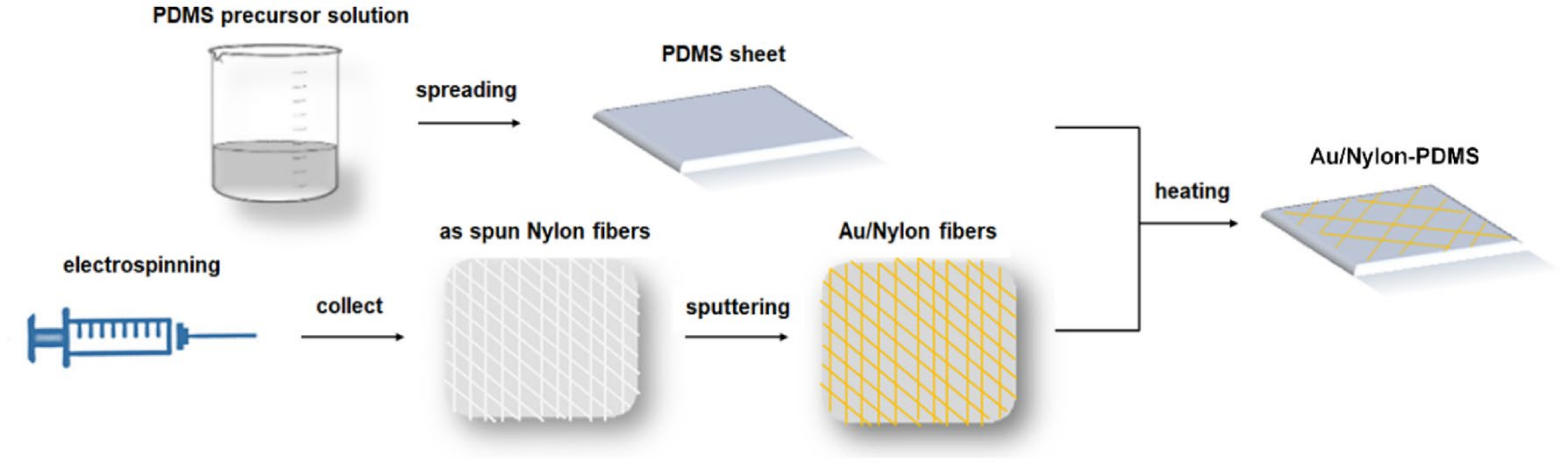
Artificial muscles fabrication steps.

The actuator manufacturing process involves three major steps (Scheme 1), namely the preparation of PDMS sheets, the fabrication of metalized electrospun fiber networks, followed by the coupling of those two components. Thus, the commercial PDMS precursor prepolymer and crosslinking agent were mixed at a ratio of 10:1 (w/w), degassed, the obtained solution being spread in thin sheets using a Byko Drive equipment. The pre-polymerized sheets were heated at 70 °C for 30 min to obtain a cross-linked polymer.

The fiber networks were prepared by electrospinning in default conditions, employing a nylon 6/6 in formic acid solution with a concentration of 22% (w/w). The electrospinning parameters were the applied voltage 25 kV, the distance between spinneret and collector 15 cm, the pumped solution flow rate 0.2 mL/h. Self-supporting fiber networks were collected on copper wire frames. The average diameter of the fibers was evaluated from SEM images to be 2.08 ± 0.1 μm. As an intermediate step, the electrospun fiber webs attached on copper frames were covered with a thin Au layer by radio frequency magnetron sputtering considering as deposition parameters: the pressure in the chamber 5.4 10^−3^ mbar, the power applied on the magnetron 30 W, and using an Au target in Ar atmosphere while the deposition time was 2 h. Gold was chosen for covering the electrospun fiber meshes in order to make them conductive and consequently enabling the low current, low voltage actuation. Gold is also flexible while presenting a high resistance to oxidation and corrosion, being a good choice for the particular application. In the last step of the fabrication process, the PDMS sheets were initially placed on a heating plate set at 120 °C for five minutes, in order to generate more flexible sticky substrates. After that, the metalized fiber network was attached to the warm PDMS sheet and the assembly was heated at 120 °C for another 30 min with compression from above. The approximate total thickness of the device was evaluated by employing the cross section SEM images and it is about 250 ± 2.5 μm. Further, the fabricated material was cut in various shapes to test its functionality.

The obtained electrospun fiber networks were morphologically characterized using a Zeiss EVO 50 scanning electron microscope (SEM) before and after 500 applied *on/off* cycles. The 0.5, 1, 1.5, 2 and 2.2 V voltages were applied using a DC source contacting the branches of the V-shaped actuator. The temperature of the actuator achieved at specified applied voltages was measured using an FLIR A305sc infrared camera with a resolution detector of 320 × 240 pixels and ResearchIR software. The digital photos and movies demonstrating the device actuation were recorded using a Canon DS126201 professional camera. Thermal properties of PDMS and Au/Nylon-PDMS samples were investigated using a LFA 457 microflash thermal properties analyzer from Netzsch with a standard SiC 12.7 mm diameter sample holder for the perpendicular to plane measurements and a special sample holder made of stainless steel for in plane measurements on 20 mm diameter samples. In the through plane setup, a Nd-YAG laser pulse of ~ 3 ms is projected on the bottom surface of the sample and the temperature evolution is recorded with an InSb IR liquid nitrogen cooled detector on the opposite top surface of the sample. In the in-plane setup, the laser pulse is applied on the middle of the bottom surface of the sample and the temperature excursion is recorded on the opposite site on 4 windows placed at a precise distance on a concentric ring outside the middle illuminated area. All measurements were made in 0.1 mbar Ar atmosphere with a high laser voltage (2690 V). The calibration needed for specific heat was made using a Ti 100 μm foil which was in turn calibrated with the standard sapphire sample from Netzsch. To highlight the ability of the PDMS-based artificial muscles to move through electromagnetic induced heating the Au/Nylon-PDMS was placed inside the winding of a standard radio frequency magnetic induction heater. For this experiment the frequency of the magnetic field was 232 kHz and the applied current to the winding was varied between 400 and 550 A.

## Data and availability

Experimental data is freely available to readers upon request.

## Supplementary Information

Supplementary Video 1.

Supplementary Video 2.
